# Development of a green binder system for paper products

**DOI:** 10.1186/1472-6750-13-28

**Published:** 2013-03-26

**Authors:** Ashley R Flory, Deborah Vicuna Requesens, Shivakumar P Devaiah, Keat Thomas Teoh, Shawn D Mansfield, Elizabeth E Hood

**Affiliations:** 1 Arkansas Biosciences Institute, Arkansas State University, Jonesboro, AR, 72467, USA; 2 Department of Biology, Arkansas State University, Jonesboro, AR, 72467, USA; 3 College of Agriculture and Technology, Arkansas State University, Jonesboro, AR, 72467, USA; 4 Department of Wood Science, University of British Columbia, 4030-2424 Main Mall, Vancouver, BC, V6T 1Z4, Canada

**Keywords:** Paper industry, Binders, Enzymes, Plant-produced proteins, Green chemistry

## Abstract

**Background:**

It is important for industries to find green chemistries for manufacturing their products that have utility, are cost-effective and that protect the environment. The paper industry is no exception. Renewable resources derived from plant components could be an excellent substitute for the chemicals that are currently used as paper binders. Air laid pressed paper products that are typically used in wet wipes must be bound together so they can resist mechanical tearing during storage and use. The binders must be strong but cost-effective. Although chemical binders are approved by the Environmental Protection Agency, the public is demanding products with lower carbon footprints and that are derived from renewable sources.

**Results:**

In this project, carbohydrates, proteins and phenolic compounds were applied to air laid, pressed paper products in order to identify potential renewable green binders that are as strong as the current commercial binders, while being organic and renewable. Each potential green binder was applied to several filter paper strips and tested for strength in the direction perpendicular to the cellulose fibril orientation. Out of the twenty binders surveyed, soy protein, gelatin, zein protein, pectin and *Salix* lignin provided comparable strength results to a currently employed chemical binder.

**Conclusions:**

These organic and renewable binders can be purchased in large quantities at low cost, require minimal reaction time and do not form viscous solutions that would clog sprayers, characteristics that make them attractive to the non-woven paper industry. As with any new process, a large-scale trial must be conducted along with an economic analysis of the procedure. However, because multiple examples of “green” binders were found that showed strong cross-linking activity, a candidate for commercial application will likely be found.

## Background

Globally, paper companies apply chemical binders during the paper-making process to attain target tensile strength of paper. Some components of these binders are acrylamide, acetaldehyde, urea-formaldehyde and vinyl acetate. Alternatives to synthetic paper binders have been investigated, but a renewable binder that can equal the strength of the traditional binders has yet to be identified. Plant-based products would be an ideal alternative to chemicals currently used in the paper industry.

Polymerized plant cell wall components result in a remarkably strong structure in nature. Since paper is made primarily of one of those polymers, cellulose, it is possible to simulate a cell wall assembly by applying and cross-linking other cell wall constituents with enzymes, such as laccase and peroxidases, which are responsible for this cross-linking *in vivo*. The ensuing interwoven network of substrates may increase paper tensile strength and provide an alternative to chemical binders. In this work, proteins, carbohydrates, and phenolic compounds were applied to paper with and without enzyme activation to determine if an increase in tensile strength could be achieved.

Phenolic compounds are the first and largest category of the three main plant-derived substrates that could potentially be utilized to create a strong binder for paper. They are made up of aromatic carbon rings with hydroxyl groups attached, and are a major component of the secondary cell wall. Lignin is a complex phenolic polymer whose composition can change depending on what precursors are involved in its assembly [1]. A few of the precursors to lignin are ferulic acid, and sinapyl and coniferyl alcohol. These compounds can cross-link to themselves, to each other, or to a growing lignin polymer with the use of laccase [2].

Black liquor is a by-product from the pulp and paper industry generated when wood pulp is chemically digested and bleached to remove lignin. Bleaching not only results in the removal of lignin, but also ferulic acid, cellulose, and hemicelluloses [3]. Because black liquor is a by-product generally used for heat energy, but rich in potentially valuable chemicals, it provides an inexpensive source of cell wall substrates. Since many of the substrates found in black liquor are phenolic compounds, this project sought to employ black liquor as a cross-linking agent in paper manufacture. However, black liquor obtained from different sources can have varying properties, depending on feedstock stream and pulping process employed at the pulp mill, and consequently is compositionally different.

Elegir *et al.*[3] previously described an experiment that attempted to use phenolic compounds as paper binders. This group isolated lignin from black liquor and cross-linked it with the use of laccase and 2,2'-azino-bis(3-ethylbenzothiazoline-6-sulphonic acid) (ABTS) in an attempt to increase the tensile strength of hot pressed pulp sheets. The authors found that the cross-linking of lignin provided more than a two-fold increase in wet tensile strength of the pulp sheet. However, dry tensile strength was negatively affected. As an alternative approach, Mansfield [4] demonstrated that directly impregnating radiate pine with laccase during mechanical pulp production could not only reduce the refining energy required to attain a target freeness, but also had enhanced paper strength properties. This strategy takes advantage of the cross-linking capacity of laccase to enhance the inter-fiber bonding using the native wood fiber lignin remaining on the pulp fibers.

Some cross-linking experiments have been performed simply to understand mechanisms by which cell wall enzymes and substrates interact. Iiyama *et al.*[2] describe mechanisms for covalent cross-linking of polysaccharides, proteins, phenolic compounds, and hydroxycinnamic acids in cell walls, but any applications of these results was not discussed. Given that the mechanisms and compounds involved in these chemical reactions are understood, these processes may be replicated *in vitro* and then applied in the paper industry as a renewable, green paper binder.

The second type of substrate predicted to be a strong binder in these experiments was protein. Many types of protein have already been utilized for making paper binders. Fahmy *et al.*[5] describe the use of plant proteins from soybean and wheat as binders in cellulosic paper composites. In their experiments, they used the total protein found in soybeans and wheat protein (gluten) to make a slurry with water, urea, sodium hydroxide, and acrylamide. They found that wheat gluten increased paper strength as much as 60%, while total soy protein increased the paper strength by 46% compared to paper with no binder added. The authors concluded that although gluten was more effective at increasing tear strength of paper, soy protein would be better suited as a bulk binder because it is less expensive than gluten. While these proteins did not provide enough increase in tensile strength to replace the current chemical binders, it is possible to decrease the amount of synthetic binder used by supplementing the industrial binder with this plant-derived protein binder. In the current project, we isolated soy proteins from defatted soybean meal, which were applied to paper, with and without enzyme, to determine if a significant increase in tensile strength would be possible.

Another protein of interest was hydroxyproline-rich glycoproteins (HRGP), which are highly glycosylated proteins found in the cell wall that contain repeated sequences of serine and proline, as well as an abundance of the amino acid tyrosine [6]. Peroxidase, which requires hydrogen peroxide to facilitate its reactions, is responsible for moving electrons so that tyrosine can attach to other tyrosine residues resulting in cross-linked HRGP in the cell wall [7]. Since this protein has been shown to cross-link in a peroxide-mediated fashion, it was also a suitable candidate as a paper binder.

Zein is an alcohol-soluble storage protein found in maize seed that is known as an excellent film-maker because of its ability to cross-link. Kim *et al.*[8] report that reagents such as glutaraldehyde, epicholorhydrin and citric acid, induce cross-linking between zein molecules. An increase in tensile strength was reported in zein films after cross-linking occurred. These properties of zein protein suggested it could be an organic, renewable alternative for a paper binder.

Although plant components are excellent choices for paper binders, some animal proteins may also serve the same purpose. Gelatin is a denatured form of collagen that is derived mainly from skin and bone of bovine and porcine sources (http://www.rousselot.com/en/rousselot-gelatine/gelatine-characteristics/definitions/gelatine-bloom/). Gelatin is tested and graded according to its bloom strength. Bloom strength is defined as the force, expressed in grams, necessary to depress a 6.67% gel (kept 17 hours at 10°C) by 4 mm with a standard plunger. Bloom strength generally ranges between 50 to 300 g and a higher bloom means stronger, usually more expensive gelatin. Gelatin is similar to HRGP in that it is inherently rich in hydroxyproline residues, thus it has side chains available for cross-linking with peroxidase, and possesses a native conformation that will allow it to lay flat against the cellulose of paper. While this protein may provide adequate tensile strength, there may be problems implementing it in a commercial setting because of ethical and health concerns about using animal products.

The last group of potential binders is carbohydrates. Carbohydrates are the most abundant class of organic compounds found in living organisms, vegetal and animal indistinctively. In this work, keeping within the scope of developing a green binder, we chose to test carbohydrates derived from plant sources, algae or fruit. Since these potential binders are made up of polysaccharides, laccase and peroxidase will not effectively cross-link these substrates. However, the experiment performed by Fahmy *et al.*[5] with soy protein and wheat gluten suggests that enzymatic cross-linking of substrates may not be necessary to produce a strong binder.

We aimed to produce a renewable binder to replace chemical binders currently used by the paper industry in order to increase tensile strength of non-woven papers. We studied the potential of oxidoreductase enzymes to cross-link substrates and produce an interwoven network of substrates within the cellulose of paper, thereby increasing tensile strength. Alternatively, these molecules may cross-link without added enzymes. In order to replace the chemical binder with an organic binder, the strength of paper had to be at least equal to that of the paper with a chemical binder. We found several potential binder compounds that produced adequate strength.

## Results

Our approach was to test a variety of binders and conditions on 1 × 0.5 inch strips of Whatman #1 filter paper. Binders were applied by submersion into solution, the paper was dried 10 minutes at high temperature and the strength was tested in the direction perpendicular to the orientation of the cellulose fibers, as this is the weakest direction and the standard in the industry. In some cases enzymes were used. To determine the utility of the enzymes, they were first tested with substrates without the paper strips to determine optimal reaction conditions. A list of all potential binders tested is found in Table 1. Tear weights for all groups of green binders were statistically tested for differences from the commercial binder control (Table 2). We were interested in binders that were as strong or stronger than the commercial binder.

**Table 1:** Summary of all substrates tested as binder

**Substrate category**	**Substrate**	**Source**
Protein	Soy Protein	Defatted soybean meal (Arkansas State University)
	HRGP	Corn silk (Arkansas State University)
	Gelatin	Knox Gelatin (Kraft Foods, Glenview, IL)
		JELL-O (Kraft Foods, Glenview, IL)
		Bovine & Porcine (Great Lakes Gelatin, Grayslake, IL)
	Zein	Acros Organics, Geel, Belgium
Carbohydrates	Agar	Seng Huad Limited Partnership (Bangkok, Thailand)
	Agarose	Genetic Analysis (Fisher Scientific, Pittsburg, PA)
		Analytical Grade (Promega, Madison, WI)
	Pectin	Sure-Jell (Kraft Foods, Glenview, IL)
		Ball (Jarden Home Brands, Daleville, IN)
		Apple (Sigma Chemical Company, St. Louis, MO)
		Grapefruit (Source Naturals, Inc., Scotts Valley, CA)
	Gum Arabic	Sigma Chemical Company, St. Louis, MO
	Xanthan Gum	Kountry Kupboard, Jonesboro, AR
	Locust Bean Gum	Sigma Chemical Company, St. Louis, MO
	Carrageen	Sigma Chemical Company, St. Louis, MO
	Kelp Powder	Now Foods, Bloomingdale, IL
	Flaxseed	Now Foods, Bloomingdale, IL
Lignin/	Black Liquor	Buckeye Technologies Inc., Memphis, TN
Phenolic Compounds		
	Lignin Low Sulfonate	Sigma Chemical Company, St. Louis, MO
	Sodium Lignin Sulfonate	MP Biomedicals, LLC, Solon, OH
	Salix	Vertichem, Toronto, Canada
	Marasperse	Lignotech, Rothschild, WI
	Ferulic Acid	Sigma Chemical Company, St. Louis, MO
	Coniferyl Alcohol	Sigma Chemical Company, St. Louis, MO

**Table 2:** Binder differences from the commercial binder

**Binder**	**Equal to commercial binder**^**1**^	**Higher than commercial binder**
**Proteins**		
HRGP + 300 ug HRP	No	
3% Zein	**Yes**	
5% Zein	**Yes**	
TSP (Pellet Resuspended)	**Yes**	
TSP/ 250 μg HRP (10 min)	No	
9% Knox Gelatin	**Yes**	
9% Knox Gelatin + 200 ug HRP	No	**Yes**
9% JELL-O	**Yes**	
Porcine 300 Bloom	**Yes**	
Bovine 250 Bloom	**Yes**	
**Carbohydrates**		
0.8% Agar	No	
3% Agar (Analytical)	No	
1% Agar (Genetic Analysis)	No	
11% Sure-Jell (low sugar)	No	
9% Sure- Jell	No	
1% Gum Arabic	No	
5% GA + 250 ug HRP	No	
Kelp	No	
Flax Seed	No	
Xanthan Gum	No	
Locust Bean Gum	No	
1% Carrageen	No	
**Pectins**		
5% Ball Pectin	**Yes**	
7% Ball Pectin	**Yes**	
9% Ball Pectin	**Yes**	
5% Apple	No	
5% Apple + 1N HCl	No	
5% Apple+CA (0.50 g)	**Yes**	
3% Grapefruit	No	
3% Grapefruit + CA (0.23 g)	**Yes**	
**Lignins and Phenolics**		
Black Liquor (Heat)	No	
Black Liquor + HCl	No	
Lignin Low Sulfonate (Heat)	No	
Lignin Low Sulfonate + HCl	No	
5% Salix (Heat)	**Yes**	
Lignin Sulfonate (Heat)	No	
Lignin Sulfonate + HCl	No	
Marasperse (Heat)	No	
Marasperse + HCl	No	
Ferulic Acid (2 mg/ml)	No	
Coniferyl Alcohol (2 mg/ml)	No	

The goal of these experiments was to find a green binder that was not significantly lower than the currently used commercial binder. All binders that were not significantly different at the 95% confidence level are marked “Yes”. Only the Knox gelatin binders were significantly higher than the commercial binder.

^1^Samples that are not significantly different are those that perform as well as the commercial binder. All samples that are significantly different have lower values except for the gelatin, which was significantly higher. See Materials and Methods for statistical treatments.

### Enzyme reactions

**Figure 1:**
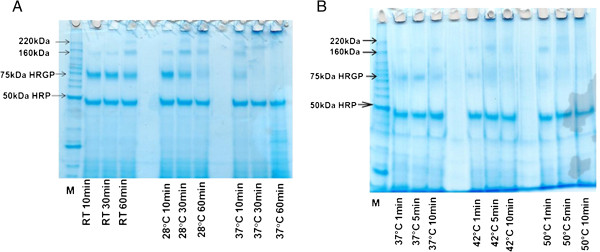
SDS-PAGE of HRGP and HRP reactions, A. RT to 37°C; B. 37°C to 50°C.

Two enzymes were tested in this study, horseradish peroxidase (HRP) and laccase. Protein gels were used to determine HRP’s optimum conditions in cross-linking proteins. Figure 1A shows reactions incubated for 10, 30 and 60 minutes at 22, 28 and 37°C. HRP is approximately 50 kDa is size, whereas the substrate, hydroxyproline-rich glycoprotein, or HRGP extracted from maize silk, is approximately 75 kDa. As heat and reaction time were increased, the band corresponding to HRGP at 75 kDa lessens in intensity and the bands at 160 kDa and those above 220 kDa become more intense. Gel electrophoresis results with HRP showed that the increase in HRGP’s molecular weight occurred most efficiently at 50°C after 1 minute of reaction using water as a buffer (pH approximately 7; Figure 1B). Figure 1B shows reactions that were incubated at 37, 42 and 50°C for 1, 5 and 10 minutes. The same concentration of substrate and enzyme was loaded on both gels shown in Figure 1A and B. The band corresponding to HRGP at 75 kDa is clearly seen at room temperature and 28°C in Figure 1A. This band is fainter and eventually disappears as the temperature increases to 50°C.

**Figure 2:**
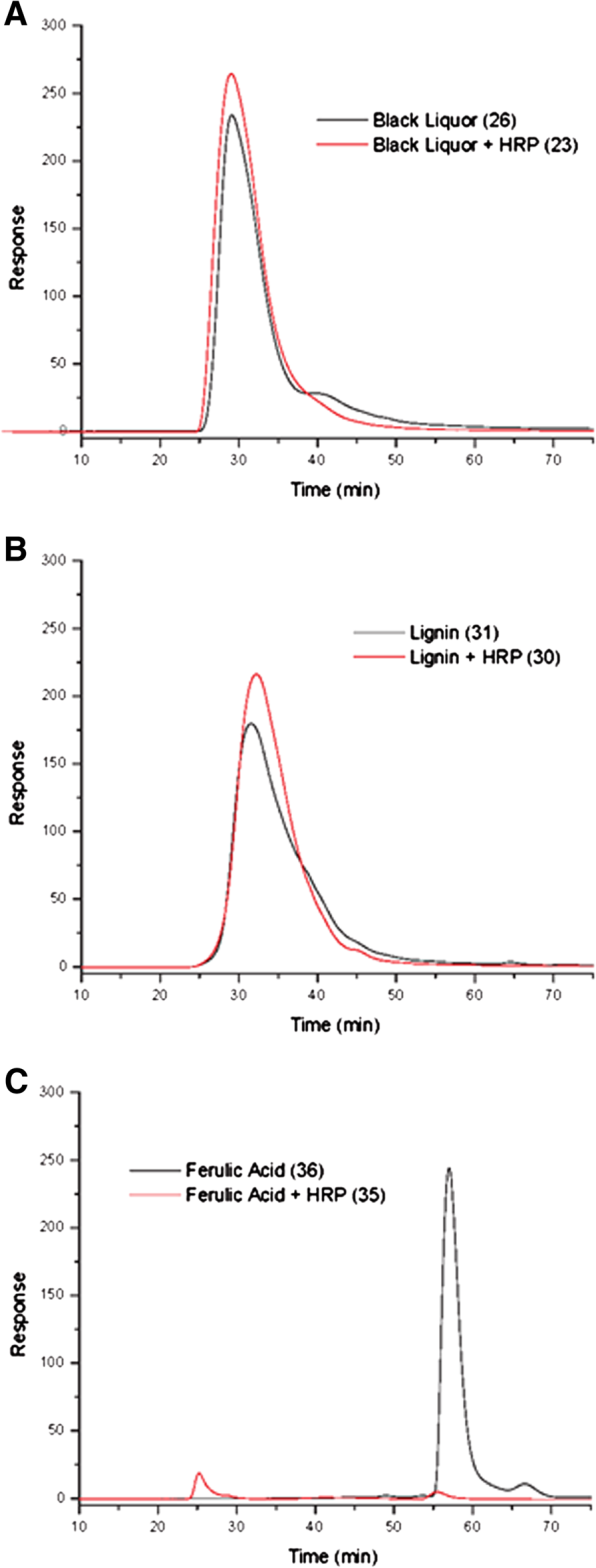
Gel permeation chromatography of various substrates with HRP.

In order to determine the efficacy of HRP on phenolic compounds, samples were analyzed by gel permeation chromatography. Low and high concentrations of HRP (15 μg and 150 μg) were combined separately with black liquor, lignin and ferulic acid before chromatographic analysis. Figure 2 shows a dramatic change in molecular weight when HRP is added to ferulic acid compared to when ferulic acid was analyzed alone. Only slight increases in molecular weight were observed in black liquor and lignin when HRP was added to these samples.

**Figure 3:**
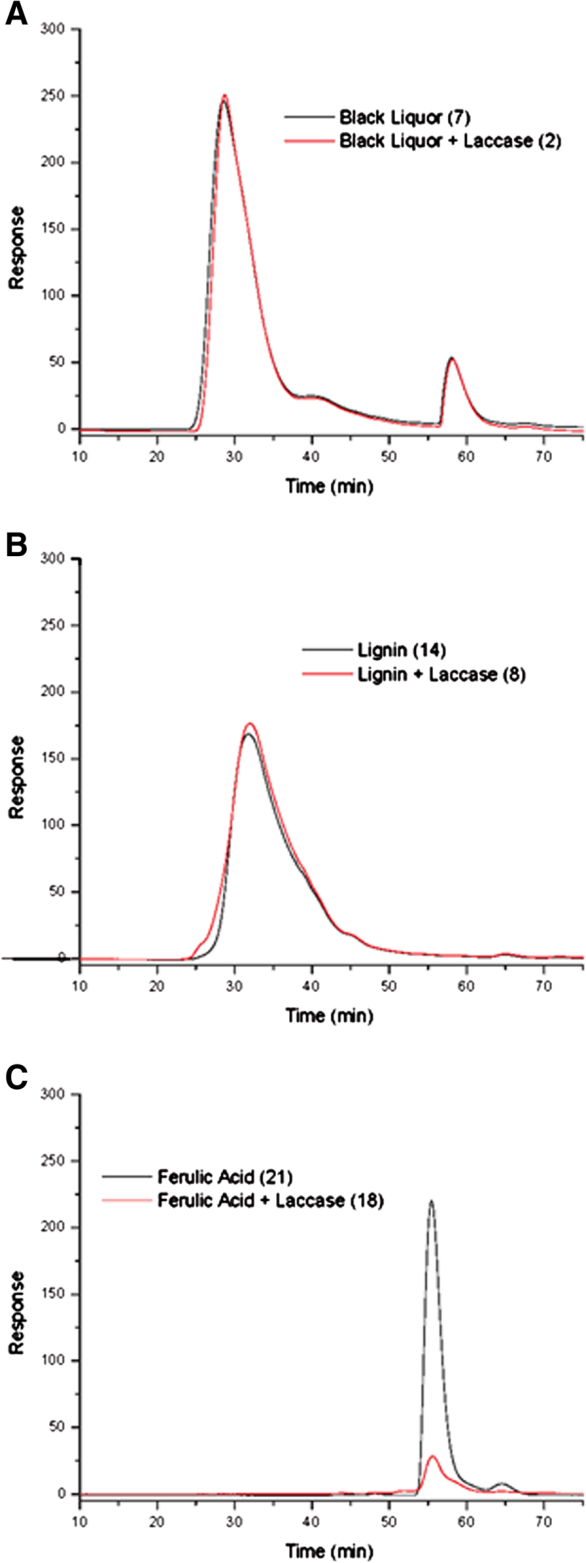
Gel permeation chromatography of various substrates with laccase.

In order to test for activity of laccase on phenolic compounds, this enzyme was combined separately with black liquor, low sulfonate lignin and ferulic acid. It was then analyzed for molecular weight changes by gel permeation chromatography. Figure 3 shows that when lignin and laccase were combined, lignin eluted earlier than when lignin was run by itself, demonstrating an increase in molecular weight of the lignin stemming from laccase cross-linking. These results also indicate there was no change in molecular weight when laccase was combined with black liquor and only a slight change when this enzyme was combined with ferulic acid under the conditions tested. The peaks of ferulic acid in Figures 2 and 3 vary because conditions for GPC detection were adjusted for the second compound in the mix rather than the ferulic acid and are thus are not quantitative.

### Protein binders

**Figure 4:**
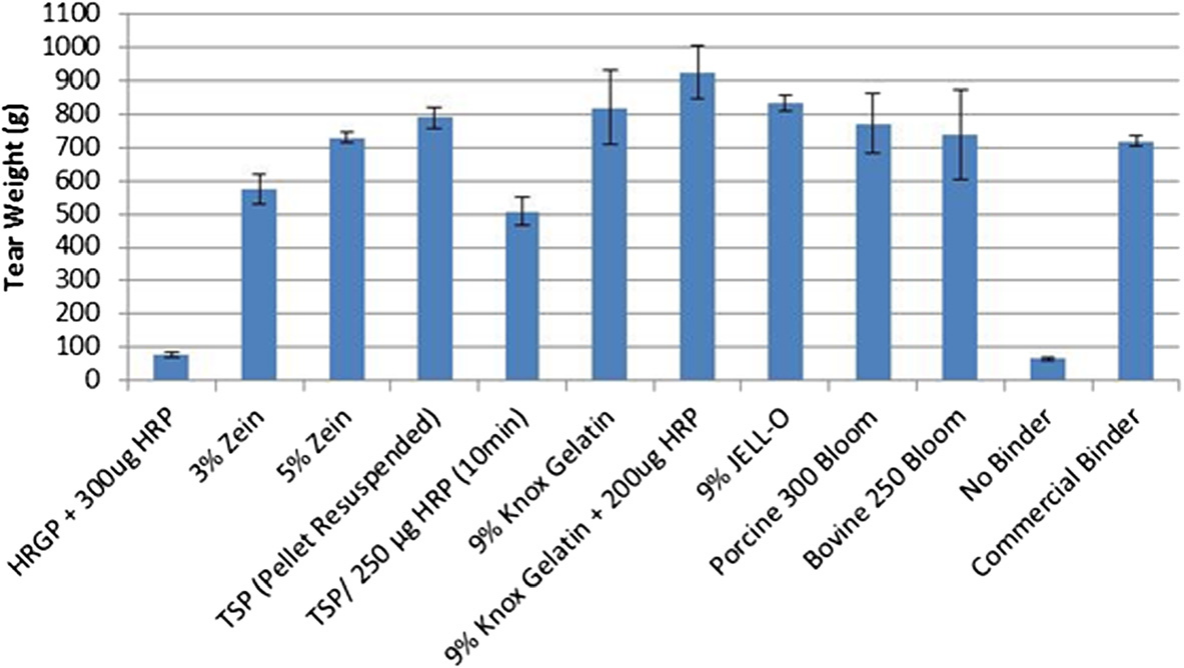
**Summary of average tensile strength of protein binders.** Tear weight is the average of the weight required to tear three strips of paper individually. Error bars represent standard deviation. HRP: Horseradish peroxidase. HRGP: Hydroxyproline-rich glycoprotein. TSP: Textured soy protein.

Several proteins were tested as binders using small paper strips (Figure 4). The units on the y-axis show the weight (in grams) necessary to tear the paper strips. Control strips of paper were received from a paper manufacturer with their vinyl acetate binder already applied. The column labeled “Commercial Binder” shows the weight needed to tear paper with the industrial binder applied. The column labeled “No Binder” refers to the average weight needed to tear three pieces of wet paper in the cross direction with no binder applied. For each binder several concentrations of enzyme and substrate were tested, as well as different incubation times with the various enzyme preparations. Strength values for selected experiments are shown.

Knox gelatin with HRP was able to withstand 925 grams of weight before the paper tore, a significantly higher tear weight (95% confidence level) compared to the commercial binder which tore at 720 grams weight. Even without enzyme, the Knox gelatin binder was able to withstand 819 g before tearing which was not different from the commercial binder. Textured soy protein held slightly less weight than gelatin (790 g), but still outperformed the commercial binder. Zein protein served as an average binder withstanding 574 grams of weight. Zein and TSP were not significantly different from the commercial binder. The weakest protein binder was HRGP, which was only able to hold about 77 g before the paper tore.

### Carbohydrate binders

**Figure 5:**
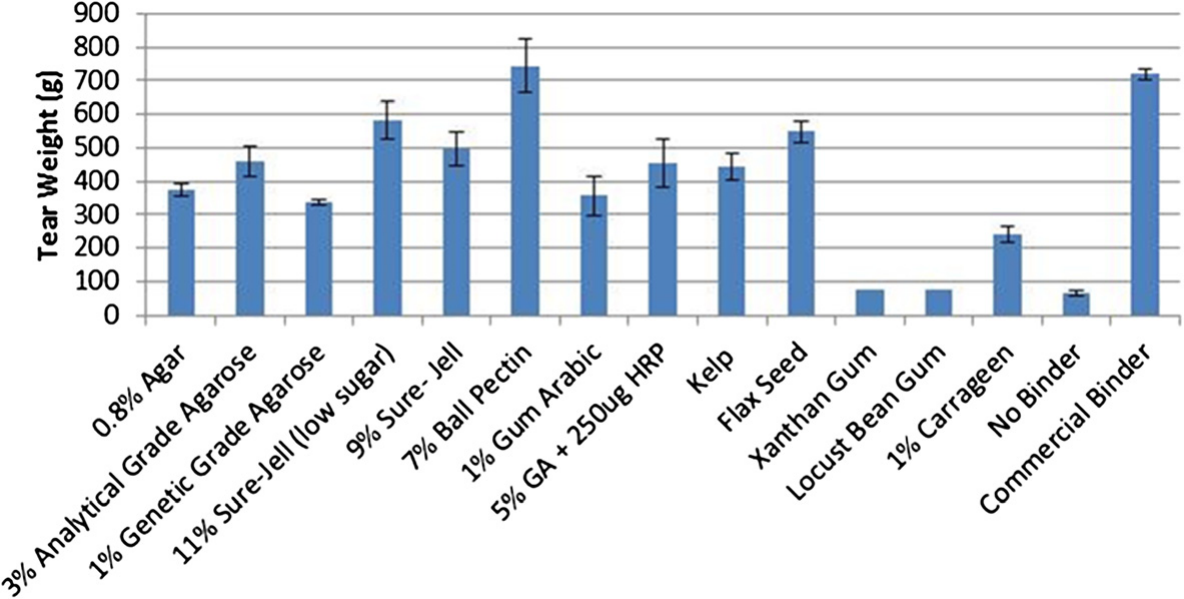
**Summary of average tensile strength of carbohydrate binders.** Tear weight is the average of the weight required to tear three strips of paper individually. Error bars represent standard deviation. GA: Gum arabic. HRP: Horseradish peroxidase.

Several carbohydrates were also used as substrates in this study (Figure 5). Results show the concentration of substrate that produced the strongest binding strength. Each vertical bar is an average of the weight it took to tear three individual pieces of wet paper in the cross direction. All carbohydrate binders were dissolved in boiling water prior to application to the paper strips. Enzymes were only used when working with gum arabic since this polysaccharide also contains some gylcoprotein [9].

Strength results from agar and two different grades of agarose (analytical grade and genetic analysis grade – see Table 1) are shown in Figure 5. These binders were able to withstand between 337 and 458 g of weight. Ball pectin’s average tear weight (745 g) was slightly higher than that of the commercial binder but not significantly different.

Regular Sure-Jell® pectin was the weakest of the three pectins evaluated holding only 495 g, while low sugar Sure-Jell was intermediate, withstanding 584 g of weight. Xanthan gum and locust bean gum binders resulted in no extra strength added to the paper. Carrageen also served as a very weak binder only holding 243 g of weight compared to the commercial binder. Brown flax seed was ground with liquid nitrogen, then mixed with water and applied to paper, and was able to hold 548 g of weight. These carbohydrate binders were all significantly lower than the commercial binder.

**Figure 6:**
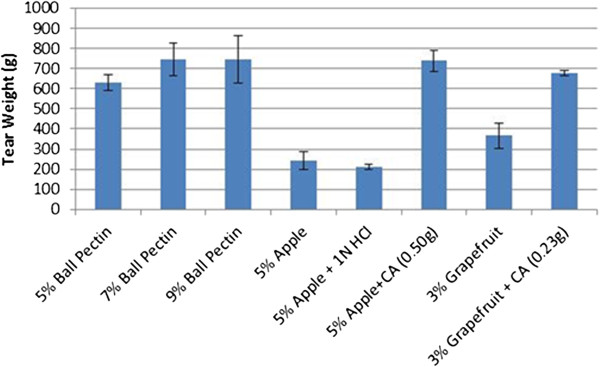
**Average tensile strength of pectin binders.** Tear weight is the average of the weight required to tear three strips of paper individually. Error bars represent standard deviation. CA: Citric Acid.

As shown in Figure 6, pure pectin (apple and grapefruit) applied to paper resulted in weak binding, only holding between 230 and 360 g of weight. However, when an acidic component such as citric acid was added, the binder was capable of withstanding between 680 and 740 g, respectively, allowing pectin to equal that of the commercial binder. Pectin concentration was also shown to affect the binder strength, e.g., 5% pectin holds about 100 g less weight than 7 or 9% pectin, although this amount is still not significantly lower than the commercial binder.

### Phenolic binders

**Figure 7:**
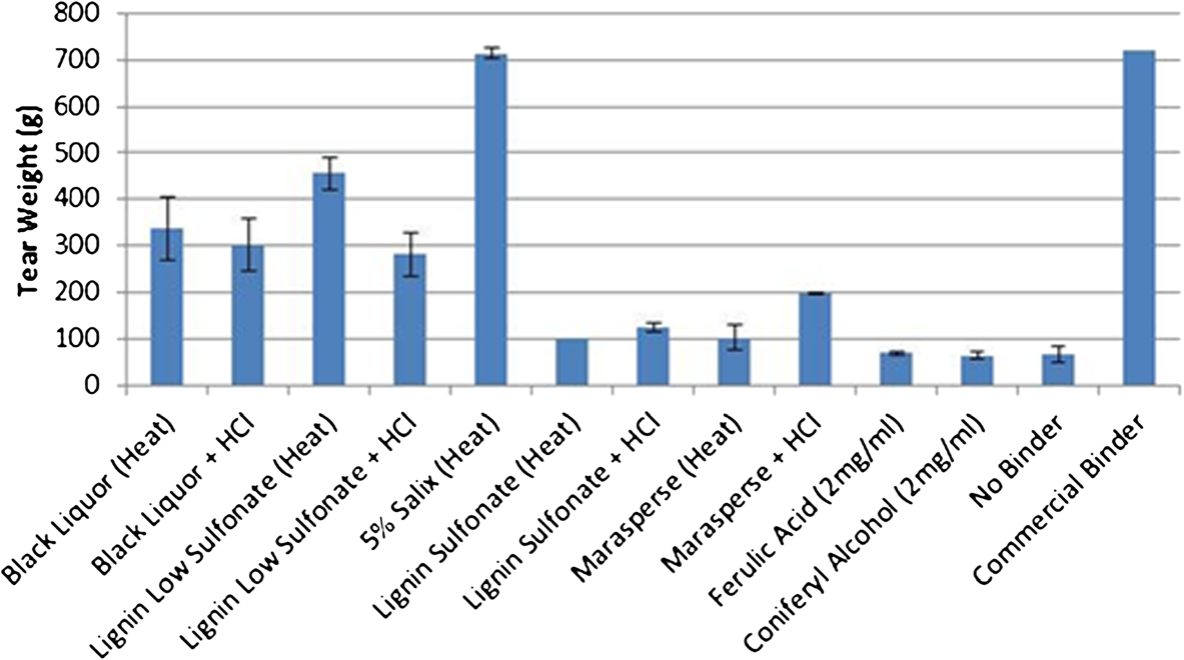
**Average tensile strength of lignin/phenolic compound binders.** Error bars represent standard deviation. HCl: Hydrochloric acid HRP: Horseradish peroxidase.

Finally, lignin and phenolic compounds were tested as potential binders. For each type of lignin applied to the paper strips, two types of experiments were carried out: lignin combined with hydrochloric acid or lignin alone, dried at high temperatures, between 100°C and 200°C. Figure 7 shows that lignin sulfonate and marasperse were only able to hold 103 g weight when high heat was applied. However, black liquor and lignin low sulfonate were able to withstand 337 and 456 g, respectively, when they were dried at 200°C. When 1N HCl was sprayed onto lignin coated paper strips, lignin sulfonate and marasperse were able to hold 123 and 198 g, respectively. The black liquor binder held 290 g and lignin low sulfonate 282 g after being sprayed with HCl. Neither coniferyl alcohol nor ferulic acid was capable of holding much weight, tearing at 65 g. *Salix* lignin when dried at 200°C was the strongest lignin/phenolic compound binder, holding 714 g, the only phenolic binder that was equal to the commercial binder. The *Salix* lignin comprises an alcohol soluble fraction from ground wood that contains complex polymerized forms of lignin (USP# 8,053,566).

### Baby wipe solution test

In order to determine the efficacy of our experiments for commercial applications, we tested our bound paper soaked in baby wipe solution over a 3 month period. Baby wipe solution tests were performed on paper treated with the ball pectin binder exclusively since this was the strongest green binder from a non-animal source tested in previous experiments. These values were compared to strength values produced by a vinyl acetate binder currently in use. We observed no significant differences between the chemical binder and the Ball pectin binder used in this experiment, before application of the baby wipe solution or after 3 months soaking in the solution (data not shown).

## Discussion

Several proteins, carbohydrates, and lignins were tested as paper binders to explore the possibility of replacing chemical binders currently used by manufacturers of non-woven paper products with an alternative green, renewable compound. Horseradish peroxidase and laccase were combined with green binders in order to initiate cross-linking between these molecules. We tested environmentally friendly binders that could be implemented into a commercial setting while still providing the same strength as the commonly used vinyl acetate binder.

In order to determine optimum conditions for the enzymes, cross-linking tests were performed by using molecular weight determination of cross-linked proteins with SDS-PAGE, or gel permeation chromatography for cross-linked lignins. Protein gels showed that HRP cross-linked HRGP, increasing its molecular weight. Otte and Barz [10] conducted a similar experiment to investigate if cross-linking was occurring between an extensin-like protein and a proline-rich protein extracted from chickpea cell walls. The authors performed SDS-PAGE analysis to demonstrate the formation of one large band above 205 kDa from two protein bands of 60 and 190 kDa when treated with HRP and H_2_O_2_, similar to the results shown in the present work. Therefore, it can be concluded that cross-linking of HRGP is occurring, as described by Deepak *et al.*[11].

Gel permeation chromatography (GPC) was used to establish conditions for reactions using phenolic compounds. GPC is commonly used to distinguish differences in molecular weight when trying to determine if cross-linking is occurring using oxidoreductase enzymes and phenolic compounds as substrates. Felby *et al.*[12] described an experiment that used laccase to cross-link lignin in order to create stronger fiberboards. The authors used GPC to compare molecular weight of lignin extracted from pulp and fiberboards to lignin from each source treated with laccase. In a similar experiment, Felby *et al.*[13] showed an increase in strength properties of beech wood fiberboards when laccase was used compared to boards that did not have the enzyme treatment.

In the present work, GPC results showed little to no change in molecular weight when enzymes were added to substrates, with the exception of ferulic acid and HRP which showed a dramatic increase in molecular weight. Because laccase was shown to have such a minimal effect on its substrates, its use was minimized in binder strength tests on paper. Also, because of the GPC results, HRP was primarily used with proteins and not with lignin or other phenolic compounds.

Lignin samples were analyzed with GPC and preliminary results led us to predict changes could be made to improve efficiency of laccase reaction conditions. Even though pH 5 sodium acetate buffer was used in all reactions as suggested by Bailey *et al.*[14], this buffer was not able to establish an ideal pH for the reaction to occur, as the lignin is extremely basic in nature. Black liquor has an especially high pH of 10–12 and the sodium acetate was only able to maintain the reaction conditions to approximately pH 8. Madzak *et al.*[15] showed that laccase has an activity range of pH 2 to 6.

Also, we predict supplementing the reaction with oxygen would improve the efficacy of the laccase reaction. Laccase works by removing electrons from oxygen, transferring them to its substrates, thereby creating free radicals or reactive species that will cross-link with each other [16]. All reactions analyzed by GPC were carried out in closed 1.5 ml Eppendorf tubes. Although these tubes were shaken during the reaction, fresh oxygen exchange could not occur. Mattinen *et al.*[17] showed the importance of oxygen in the reaction by mixing laccase and lignin together and measuring the amount of oxygen consumed over a 3 hour period with an oxygen electrode. After these solutions were shaken for 3 hours in a sealed glass flask, the oxygen concentration remaining in the flask was very low for those reactions that performed efficiently. To resolve this problem of depleted oxygen, Elegir *et al.*[3] supplemented more oxygen than was required by the reaction, ensuring oxygen would not be the limiting factor.

Mediators such as 2,2'-azino-bis(3-ethylbenzthiazoline-6-sulphonic acid) (ABTS) have also been employed to induce a conformational change in laccase that results in greater access to the enzyme’s active site. If the active site is more accessible, the enzyme should work more efficiently to cross-link substrates [2]. However, enzymes and mediators are expensive and not likely to be used in manufacturing unless substantial benefit can be gained in product quality or process efficiency.

Gelatin is a substance that is commonly used in paper sizing [18]. Paper sizing is defined as a substance applied to paper in order to provide a glossy finish that will decrease liquid absorption and improve printability and smoothness. A Sheffield test is commonly used to test the quality of paper sizing, where the longer it takes for paper to absorb a liquid, the better. Gelatin applied to paper not only improves the surface properties, but also minimizes the effect of aging and photooxidation [19]. However, it is not clear if the use of gelatin in this way has ever been shown to increase the tear strength of paper.

Gelatin could be easily implemented into the paper industry process. It is organic, renewable, readily available at low price, needs little reaction time to produce high tear strength, and produces a non-viscous solution that will not clog sprayers. Textured soy protein solution has all of these advantages as well. However, if using the resuspended pellet of soy protein, the solution may be too viscous and could clog sprayers.

In the current investigation, gelatin and textured soy protein showed the best tear strength properties of all the protein binders assessed when applied to paper, providing between a 12 and 14-fold increase in wet paper tear strength when compared to paper with no binder applied. Furthermore, the strength provided by the gelatin binder gradually increases as the percentage of gelatin increases, and is the only green binder tested that was superior to the commercial binder strength. Also, the strength provided by gelatin increases slightly when HRP is added to the reaction mixture. Gelatin from both porcine and bovine sources was tested along with a high and low bloom for each source. Neither the source of the gelatin nor the bloom strength seemed to affect the tear strength produced by these binders.

The supernatant of the textured soy protein provided approximately 50% less strength than gelatin and the use of HRP with soy protein did not seem to change this strength value. However, when the remaining pellet was resuspended in water, the wet tear strength of paper increased more than 50%, offering a product that was slightly stronger than the commercial binder product.

In theory, HRGP is an excellent substrate to be used as a paper binder. It has side chains available for cross-linking with peroxidase, which will form strong covalent bonds, and its conformation will allow it to lay flat against the cellulose of paper. There have been no previous suggestions in the literature for industrial applications of HRGP unlike the plethora of other substrates that have been evaluated as binders. However, it is known that the cross-linking of HRGP, as well as lignin and cellulose are crucial in order to provide structure and strength to the plant cell wall [2]. Therefore, if larger quantities of this protein were employed, it is reasonable to assume that an HRGP binder could provide acceptable tear strength to paper products. Nonetheless, in the present study, HRGP did not provide any extra strength to wet paper tear strength. This could very likely be due to the very low quantities of protein applied.

Zein protein from corn kernels is also a good candidate, as when zein is dissolved in ethanol and heated, it is not viscous and would not clog sprayers. Also, zein protein provided a 9-fold increase in tear strength when compared to paper with no binder applied. Because the development of low-cost manufacturing methods is needed, this protein could fit this need as it is available in large amounts. It is 40 to 50% of the protein in corn as reported by Shukla & Cheryan [20].

Carbohydrates have already been used as wood and paper/cardboard adhesives [21,22]. For example, pectin from orange peels serves as an excellent binder for drug tablets [23]. These authors also suggest that this binder would be more appealing than synthetic binders because of its availability and low cost. Coffin & Fishman [24] investigated the tensile strength and other physical and mechanical properties produced by citrus pectin films and compared those properties to films produced by sugar beet and almond pectin. Determining the differences in the properties of films made by different pectins will help to understand what type of pectin would make the best binder.

Pectin provided the highest tear strength out of all carbohydrate binders, equaling the strength produced by the commercial binder. However, there are several different types of pectin and all of them are slightly different in terms of solubility and strength provided as a binder. While all food grade pectins already contained an acidic component to assist in gelling, usually citric acid or sodium citrate, these components must be added to pure pectin. Without this added acidic component, pure pectin produced low strength values. We clearly demonstrate how adding citric acid to the pure pectin increased strength values, by up to 1.5-fold. Unfortunately, in this work, all carbohydrate binders, other than pectin either produced average to low strength results and/or their solutions were too viscous to be used in manufacturing.

Since lignin is the second most abundant polymer in nature, it can be obtained in large quantities and therefore would be suitable for a commercial setting. It was observed that when HCl was added to lignin, a sticky, gel-like mixture formed. This observation implies some form of cross-linking is occurring between the lignin molecules. Thus, we hypothesized that applying lignin to paper and then spraying that paper with HCl may have a positive effect on the strength of paper. Also, after observing an increase in strength after heat was applied to gelatin and pectin samples, it suggested that heat may have the same effect on lignin samples, as was previously reported by Mansfield [4] with mechanical pulp. Therefore, three treatments were performed on all lignin samples: addition of laccase, spraying with HCl, and application of high heat when drying. No increase in strength was apparent in any of the lignin samples when laccase was added. Slight increases in strength were observed in marasperse, black liquor, and low sulfonate lignin binders when HCl was sprayed on lignin coated paper. HCl spraying had the most effect on black liquor and low sulfonate lignin binders. High heat obviously had the greatest effect (Figure 7).

Results from zein, gelatin, pectin, soy and lignin binders show that application of heat dramatically increased the strength of paper. The possible reasons for this are as follows. First, the application of heat may simply be dehydrating the binder mix and forming a strong film around the cellulose of paper that would not be soluble in water. The literature shows that films are formed from several of the binders used in this work such as zein, pectin and gelatin [20,25,26]. These authors reported remarkable strength produced by each of these films and proposed their use in industrial applications to replace the use of petroleum products as adhesives. Another possibility is that chemical or physical changes may be occurring as a consequence of the high heat treatment, changing the flow properties of the molecules. Therefore, the strength provided by heat could be occurring because of dehydration, melting of compounds or a combination thereof.

The phenolic compounds used did not produce comparable strength values to the lignin binders. After application of ferulic acid and coniferyl alcohol, the paper tore with the same weight as did paper with no binder applied. The GPC results showed that ferulic acid had a dramatic increase in molecular weight when HRP was added, suggesting that ferulic acid could cross-link with cellulose fibers and serve as a strong binder. Unfortunately, only small amounts of ferulic acid and coniferyl alcohol could be used when applying solutions to paper because of their low solubility in water. Using ethanol as a solvent would resolve solubility, but is incompatible with enzymes.

The *Salix* binder provided the same amount of strength as the commercial vinyl acetate binder. After being dissolved in ethanol and heated, this binder forms a non-viscous solution that would not likely clog sprayers. Consequently, this binder is the most practical out of all other lignin/phenolic compound binders tested.

If black liquor provided adequate strength, it would have been the most ideal binder. Paper manufacturers produce between 250 and 400 gallons of black liquor per ton of pulp (AF&PA, http://www.afandpa.org/). Most of this is used as an energy source to avoid the use of fossil fuels, but much is excess. If paper manufacturers could use their by-product not only to fuel their mill, but also as the binder in their manufactured products, this could drastically lower production costs. As shown in the strength results for different lignin binders, slight changes in lignin composition or source of lignin can dramatically affect the strength of the binder. Therefore, in future experiments, it would be worthwhile to survey black liquor from different paper mills to determine if any samples could be implemented as a strong binder for paper products, and evaluate other reaction conditions.

Depending on the type of trees used and differences in the pulping process between facilities, the constituents of black liquor may be altered which may in turn have an effect on its properties as a binder [1]. In this work, lignin from hardwood trees showed strengths 2-fold higher than the black liquor from softwood trees. The lignins were also extracted by very different processes, perhaps contributing to the differences in binding activity. Many studies have been done to determine the structural and chemical differences between hardwood and softwood lignin. For example, Mohan *et al.*[27] found that the molecular weight of softwood pyrolysis lignin is larger than that of hardwood pyrolysis lignin. They also reported that softwood lignin which consists solely of guaiacyl-derived monomers results from high amounts of polymerized phenylpropane units, while hardwood lignin is made up of guaiacyl-syringyl lignin and results in mixed polymers of lower molecular mass.

The binders developed in this project could be applied to different commercial products, such as wet wipe applicators. Since binders produced adequate strength without the use of enzymes, covalent bonds were not formed, so it was important to determine if binders could retain their strength in commercial solutions. Therefore, paper with binder applied was soaked in baby wipe solution for up to three months before tensile strength was tested. The plant binder retained its strength just as well as the vinyl acetate binder after soaking in baby wipe solution for these periods of time.

## Conclusions

This project aimed to identify potential renewable and green replacements for the currently used synthetic paper binders. A selection of twenty binders was surveyed, and among them, soy protein, gelatin, zein protein, pectin, and *Salix* lignin provided wet tear strength equivalent to that of the vinyl acetate binder currently used in the manufacturing process. All of these binders are organic, renewable, available in large quantities at reasonable prices, require very short reaction time and do not form viscous solutions that would clog sprayers. For these reasons, all binders chosen in this work could easily be implemented in an industrial process. Oxidoreductase enzymes were combined with binder substrates to determine if enzymatic cross-linking could provide an increase in tear strength. Gelatin showed a slight increase in strength when horseradish peroxidase was added, but more research in this area is necessary to determine the true potential of these enzymes in binder systems. This effort clearly shows that there is potential for renewable resources, such as plant and animal by-products, to substitute the current level of petroleum-derived chemicals used as binders in the paper industry. Gelatin at 9% concentration combined with HRP provided significantly higher tear strength than the current commercial binder.

## Methods

### Testing method

A small-scale version of a column-testing instrument (Instron, Norwood, MA) was made in order to test the strength of paper in the laboratory setting. A die cutter was used to ensure all pieces of paper were cut exactly the same in the cross-direction. To test paper strength, the top portion of a small piece of Whatman #1 filter paper (1.5 in × 0.5 in) was attached to a holder and weight was added to the opposite end of the strip. We calculated the exact amount of weight required to tear that individual piece of paper. This process was repeated three times for each binder applied.

To test all the potential green binders, approximately 2 ml of each enzyme/substrate mixture described below was dispensed into incubator trays and the strips of paper were allowed to incubate in this solution. All reactions were incubated at 50°C and the pH used depended on the results of the enzyme tests previously performed. Reactions containing enzymes were allowed to proceed for 1 to 15 minutes.

After incubating in binder solution, the strips were dried at approximately 200°C for 10 min, submerged in water, and the strength of each wet paper strip was tested in the cross-direction. Three paper strips (i.e., 3 replicates) were used for each treatment. The mean weight needed to tear each set of three strips of paper for each treatment was recorded. These measurements were compared to two control treatments: paper with no binder applied and paper with the vinyl acetate binder applied.

### Substrate cross-linking

Horseradish peroxidase (HRP) was obtained from Sigma Chemical Co. (P6782 St. Louis, MO). Gel electrophoresis of reaction products was used to determine the extent the substrate gained molecular weight, or was cross-linked, after reacting with the enzyme under certain conditions. Thermo Scientific gradient gels (4-20% acrylamide) were used with HEPES buffer.

Approximately 6 μg of HRGP, 5 μg of HRP, and 6 μg of 8 mM hydrogen peroxide were used, as described by Devaiah & Shetty [28], and incubated at room temperature for one hour, then analyzed by SDS-PAGE. The same concentrations were used in reaction mixtures that were incubated at 22, 28, 37, 42 and 50°C, for 1, 5, 10, 30 and 60 minutes before being stopped with 0.1% SDS and mercaptoethanol and separated on a gel. The same experiment was per-formed at different pH (2, 3, 4, 5 and 6).

HRP was also combined with lignin, black liquor and ferulic acid, individually. These samples were tested with gel permeation chromatography, as described by Mansfield *et al.*[29], to determine if this enzyme caused any molecular weight changes in these phenolic compounds. Gel permeation chromatography columns, series 60 and 300, were utilized (YMC Co., Ltd. Kyoto, Japan). The column packing material was silica derivatized with 1,2-dihydroxypropane.

Lignin, black liquor and ferulic acid were each combined with laccase and gel permeation chromatography was performed. Reactions were incubated in sodium acetate buffer, pH 5, at 50°C as reported by Bailey *et al.*[14]. Samples were allowed to incubate for 15 and 60 minute time periods to ensure that cross-linking occurred. For each time period, a high and low concentration of 150 μg and 15 μg were chosen for the reaction mixture.

### Protein binders

Gelatin (Great Lakes Gelatin Co., Grayslake, IL), hydroxyproline-rich glycoprotein (HRGP, isolated from maize silk), textured soy protein (TSP, residue post oil extraction) and zein (Acros Organics, New Jersey, USA) were tested as protein binders. JELL-O® (Kraft Foods, Glenview, IL) and Knox® unflavored gelatin (Kraft Foods, Glenview, IL) were tested.

Knox® gelatin concentrations of 5, 7 and 9% were dissolved in boiling water then applied to Whatman #1 filter paper. For each percentage of Knox® gelatin used, 0, 150, 200, and 250 μg of HRP was added. All reactions were incubated for 1 minute at 50°C. Gelatin from porcine sources (blooms 100 and 300) and bovine sources (blooms 100 and 250) were obtained from Great Lakes Gelatin Company (Grayslake, IL). The highest and lowest bloom strength available from each source was purchased. For each source and for each bloom strength, 5, 7 and 9% gelatin solutions were made as above and applied to paper. The 9% JELL-O® was applied to paper with and without HRP.

HRGP was extracted from the cell walls of maize silk with calcium chloride and sodium metabisulfite as described by Hood *et al.*[6]. Concentration of HRGP was determined by absorbance at 280 nm using a microplate reader (Synergy HT, Bio-Tek). The concentration of HRGP applied to paper was approximately 1mg/ml and the reaction with HRP and H_2_O_2_ added was allowed to proceed at 50°C for 15 minutes before the paper was dried and the strength was tested.

Defatted soybean meal was ground in a coffee grinder and then mixed with water at 1:10 (w/v). The mixture of water, ground meal, HRP, and H_2_O_2_ was analyzed by protein electrophoresis to determine if cross-linking occurred with this substrate. The ground soy slurry was centrifuged for 5 minutes at 1,860 × g. The supernatant was then applied to paper strips with 250 μg of HRP and 8 mM H_2_O_2_ and was carried out for 1 and 10 minutes at 50°C. The supernatant was also applied to paper with no enzyme present. Finally, the pellet was resuspended in water and applied to paper with no enzyme present.

Zein protein was obtained from Sigma Chemical Co. (St. Louis, MO). A cross-linking test for this substrate was done by gel electrophoresis. A 3% solution of zein in 50% ethanol was applied to paper with and without HRP.

### Carbohydrate binders

A variety of carbohydrates that are common substitutes for gelatin were tested. A range of 1 to 15% of each carbohydrate was dissolved in boiling water. Since gum Arabic contains both polysaccharides and glycoproteins, a 5% solution was made with boiling water and applied to paper with 250 μg of HRP and 8mM H_2_O_2_ and without enzyme. Flax seeds were ground with liquid nitrogen and 10 ml of water was added to every gram of ground flaxseed before application to paper.

Several different types of pectin were utilized throughout this project. Two types of Sure-Jell® pectin and Ball® pectin were obtained from local grocers. The first pectin had less sugar added than the second, regular pectin. These two pectins also contained different acidic components that assist in gelling. Low sugar pectin contained fumaric acid and sodium citrate whereas the regular pectin only contained citric acid. Ball® pectin’s ingredients included citric acid, sodium citrate and potassium sorbate. Boiling water was not necessary to dissolve Ball® pectin. Sure-Jell® pectins were dissolved in boiling water.

To determine the effectiveness of pure pectins as binders, apple pectin was purchased from Sigma Chemical Co. (St. Louis, MO) and grapefruit pectin from a local health food store. For pure apple and grapefruit pectin, solutions of 1-5% were dissolved in boiling water and were applied to paper with and without citric acid. The amount of citric acid added was half the amount by weight of pectin added in each solution.

### Lignin/Phenolic compound binders

Black liquor, lignin low sulfonate, sodium lignin sulfonate, marasperse, ferulic acid, coniferyl alcohol and *Salix* lignin were the potential substrates used in these sets of experiments.

Samples of black liquor were obtained from Buckeye Technologies (Perry, FL). Ultra filtration of black liquor, using nitrogen gas and a regenerated cellulose membrane with a 1,000 Dalton molecular weight cutoff, was used to remove unwanted sulfur salts and to concentrate the sample. Approximately 5 ml of black liquor was mixed with 100 ml of water in order to ensure adequate filtration was achieved and to lessen the chance of the black liquor clogging the filter.

Three types of tests were carried out with this binder. First, black liquor was applied to Whatman #1 filter paper with and without enzymes. Three solutions were made; black liquor with 250 μg of laccase, black liquor with 250 μg of HRP and 8mM H_2_O_2,_ and black liquor with no enzyme. After applying these solutions to paper, they were dried at 100°C for 10 minutes.

Second, paper strips were dipped in black liquor, hung up with binder clips in the fume hood and 1N HCl was sprayed on both sides of the strips with a thin-layer chromatography sprayer. The strips were allowed to air dry for 5 minutes then dried completely at 100°C for 10 minutes.

Third, high heat was applied to black liquor treated paper. After liquor application on to paper, the paper strips were dried at 200°C for 10 minutes before being rewet and tested for strength in the cross-direction.

Lignin low sulfonate (Sigma Chemical Co. St. Louis, MO), sodium lignin sulfonate, (MP Biomedicals, LLC.Solon, OH) and marasperse (Lignotech Rothschild, WI) were dissolved in water to make 0.05, 0.1 and 0.15 g/ml solutions for application to paper. Each concentration of each type of lignin was applied to paper and sprayed with HCl as previously described for black liquor. HCl was added to lignin samples in order to reduce the pH of the solution [14]. These paper strips were dried at 100°C for 10 minutes. Lastly, all concentrations of each lignin were applied to paper and dried at temperatures ranging from 100°C to 200°C for 10 minutes before tensile strength was tested.

Ferulic acid and coniferyl alcohol were obtained from Sigma Chemical Co. (St. Louis, MO). Two mg of ferulic acid were first dissolved in 100 μl of dimethyl sulfoxide (DMSO), then water was added to equal 1 ml. Two mg of coniferyl alcohol were dissolved in 1ml of water. Both substrates were mixed with 250 μg of HRP and 8mM H_2_O_2_ for application on to Whatman #1 filter paper.

Finally, 0.05 g/ml and 0.10 g/ml of *Salix* lignin (Vertichem, Toronto, Canada) were dissolved in 60% ethanol. This solution was heated to approximately 55°C in the microwave to dissolve any insoluble fractions. The solution was then applied to paper, allowed to dry at room temperature for 5 minutes then dried at 200°C for 10 minutes before rewetting and testing strength in the cross-direction.

### Baby wipe solution tests

Small strips of Whatman filter paper with binder of interest applied were soaked in baby wipe solution, at room temperature for up to 3 months. The strength was tested and compared to the factory’s binder (referred to as commercial binder) that had also been soaked in solution for the same amount of time.

## Abbreviations

ABTS: 2,2'-azino-bis(3-ethylbenzothiazoline-6-sulphonic acid); DMSO: Dimethyl sulfoxide; FA: Ferulic acid; GPC: Gel permeation chromatography; HCl: Hydrochloric acid; HRGP: Hydroxyproline-rich glycoprotein; HRP: Horseradish peroxidase; kDa: KiloDaltons; PEO: Poly (ethylene) oxide; SDS-PAGE: Sodium dodecyl sulfate-polyacrylamide gel electrophoresis; TSP: Textured soy protein.

## Competing interests

The authors declare that they have no competing interests.

## Authors’ contributions

AF carried out the experiments with binders and enzyme testing. DVR coordinated, drafted and edited the manuscript. SD participated in the design of the study and performed some enzyme reactivity assays. SM performed the gel permeation chromatography. KT served as advisor throughout the project and performed the initial lignin-acid precipitation experiments. EH conceived the study, participated in its coordination and helped to draft the manuscript. All authors read and approved the final manuscript.

## Authors’ information

EEH has worked in the area of cell wall structure and function for 25 years. This project represents an application of that knowledge. SPD is an enzyme biochemist and advised the enzyme reactions. SDM is a lignin chemist and advised the lignin trials and performed the GPC.
